# Response to the ISSCR guidelines on human–animal chimera research

**DOI:** 10.1111/bioe.13104

**Published:** 2022-11-02

**Authors:** Julian J. Koplin

**Affiliations:** ^1^ Melbourne Law School University of Melbourne Melbourne Australia; ^2^ Biomedical Ethics Research Group Murdoch Children's Research Institute Melbourne Australia; ^3^ Monash Bioethics Centre Monash University Melbourne Australia

**Keywords:** chimeras, moral status, neuroethics, stem cells

## Abstract

The International Society for Stem Cell Research (ISSCR) has recently released the 2021 update of its guidelines. The update includes detailed new recommendations on human–animal chimera research. This paper argues that the ISSCR recommendations fail to address the core ethical concerns raised by neurological chimeras—namely, concerns about moral status. In minimising moral status concerns, the ISSCR both breaks rank with other major reports on human–animal chimera research and rely on controversial claims about the grounds of moral status that many people will rightly reject. A more robust framework for regulating human–animal chimera research still needs to be developed.

## 1 INTRODUCTION

In May 2021, the International Society for Stem Cell Research (ISSCR) updated its guidelines. Like previous iterations, the 2021 guidelines are intended to serve as the ‘standard for the field [of stem cell research]’ and inform the decisions of scientists, regulators, and research funders.^1^ The guidelines include detailed new recommendations on human–animal chimera research, which build on suggestions originally developed in a standalone 2007 report.^2^

Human–animal chimera research is ethically controversial, particularly in the case of experiments where human cells could contribute to animal brains. One core concern is that neurological chimeras could develop enhanced cognitive capacities; this, in turn, could arguably affect the animal's moral status (and thereby whether it is appropriate to use them in research.) Perhaps surprisingly, the ISSCR guidelines do not offer guidance for managing moral status concerns. Instead, they sketch how existing animal research standards such as the Three R's (reduce, refine, replace) can be tailored to human–animal chimeras.

This paper argues that the ISSCR's new guidelines fail to address the core ethical concerns raised by neurological chimeras. I first describe how questions of moral status have been addressed in other major reports and bioethical work on human–animal chimeras. Next, I describe the ISSCR's recommendations, focusing particularly on how they fail to address issues of moral status. Finally, I highlight and critique the philosophical basis for the ISSCR's suggestions. The philosophical assumptions underlying these suggestions have implications that many people would wish to reject. Specifically, they would license not only blanket acceptance of human–animal chimera research but also infanticide and certain forms of child neglect. The ISSCR's assumptions about moral status, therefore, provide unstable ground on which to build global stem cell policy.

## 2 EARLIER RECOMMENDATIONS REGARDING HUMAN–ANIMAL NEUROLOGICAL CHIMERAS

Human–animal neurological chimeras have many exciting potential applications, including for modelling and studying human brain diseases, testing drugs that interact with neural tissue, and potentially in regenerative medicine. Given the potential benefits of such research, there is a moral imperative not to restrict it without good reason.^3^ At the same time, the biological ‘humanisation’ of animal brains raises difficult issues of moral status—issues that many believe do justify limiting certain kinds of chimera experiments. The concern here is that ‘humanising’ an animal's brain might alter its cognition in ways that affect our moral obligations to that animal.

The term ‘moral status’ is commonly used by philosophers as a shorthand for descriptions of our moral obligations to various kinds of beings.^4^ Normal human adults are usually considered exemplars of full moral status. It is because of this moral status that we have moral obligations to treat humans respectfully and carefully consider their interests. Many theorists ground humans' moral status in our cognitive capacities, such as rationality, autonomy, the ability to engage in moral reasoning, or certain forms of self‐consciousness.^5^ If a human–animal chimera were to develop similar capacities, then its interests might deserve similar moral consideration to those of a normal human adult. This, in turn, would make it morally impermissible to use it for the kinds of experiments we subject research animals to. As Henry T. Greely has put it:

When we avoid unethical research [on humans] by making living models of human brains, we may make our models so good that they themselves deserve some of the kinds of ethical and legal respect that have hindered brain research in human beings. If it looks like a human brain and acts like a human brain, at what point do we have to treat it like a human brain—or a human being?^6^

This line of thinking is well‐established in the bioethical literature on chimeras.^7^ Moral status concerns have also featured prominently in media coverage of relevant breakthroughs,^8^ as well as major reports on the regulation of human–animal chimera research (as will be shown below).

Concerns about moral status have already posed some obstacles to human–animal chimera research. In the United States, the National Institute of Health placed a moratorium on funding certain forms of human–animal chimera research in 2015. The moratorium—which currently remains in place—was sparked by concerns that new techniques for generating chimeric animals, such as interspecies blastocyst complementation, might result in human cells contributing to the animal's nervous system and altering their cognitive state, which could ‘be problematic from ethical and animal welfare perspectives’.^9^

More recently, the U.S. National Academy of Sciences, Engineering, and Medicine (NASEM) released a report on the science, ethics, and governance of certain forms of stem cell research, including human–animal chimera research. The report notes that many capacities thought to be associated with higher moral status are ‘located’ in the brain,^10^ and notes that it is theoretically possible that human‐animal neurological chimeras could develop capacities that many people find relevant to moral status and should arguably rule out these animals' use in research.^11^ The committee did not offer specific guidance on how such concerns should be managed, since it held that such concerns are unlikely to be realised in the near future. However, the report emphasises the need for further ethical analysis and ongoing surveillance, and flags that additional oversight and restrictions might become necessary in the future.^12^ These restrictions might include designating specific categories of chimera research that should not be conducted.

In the United Kingdom, a 2011 report by the Academy of Medical Sciences discusses similar issues. This report is likewise animated by concerns that ‘humanising’ animals' brains could enhance their cognitive abilities or create human‐like consciousness or behaviour.^13^ The report raises special concern about the potential effect of such research on the cognitive abilities of larger animals such as sheep, pigs, and nonhuman primates. If ‘neural humanisation’ enhances the animal's cognition to a great enough degree—for example, if a human–monkey brain chimera develops similar cognitive capacities to a Great Ape (which are not used in research in the United Kingdom)—then normal animal research principles would seem to provide inadequate protection of humanised animals' rights and interests.^14^ The report ultimately recommends that the most ethically risky categories of human‐nonhuman chimera brain research, such as the humanisation of nonhuman primate brains, not proceed at this time. It also recommends that other research involving substantial modification of an animal's brain be subject to review by a multidisciplinary national expert body, taking into account any relevant ethical issues.^15^

These two reports are far from unique. Major reports from European countries such as Germany and Switzerland raise similar moral status concerns. Their recommendations include (in the case of Germany) introducing additional oversight of human‐nonhuman primate brain chimera research, and (in the case of Switzerland) altogether prohibiting the creation of chimeras via combining human stem cells with animal embryos.^16^ In short, while there is no consensus on *how* moral status concerns should be managed in the case of human–animal chimeras, there is widespread agreement that moral status concerns are legitimate, important, and deserving of some form of regulatory response.

## 3 ISSCR RECOMMENDATIONS REGARDING HUMAN–ANIMAL CHIMERA RESEARCH

The 2021 ISSCR Guidelines differ from the above reports and literature in a crucial sense: they are predicated on the idea that human–animal brain chimeras will not develop higher moral status than other research animals. The ISSCR's recommendations have been elaborated and explained in a recent white paper published in *Stem Cell Reports*,^17^ which supplements and expands on the guidelines.^18^ Both sources are discussed below.

The Guidelines address two forms of human–animal neurological chimera research. The first category involves the transfer of human material into the central nervous system of *postnatal* animals; the second involves the transfer of human material into animal embryos or fetuses.

The ISSCR holds that chimera studies involving postnatal animals should be exempt from specialised scientific and ethics oversight. Like other forms of animal research, they should be reviewed by existing institutional animal research committees, albeit in some cases with supplementary reviewer expertise in stem cell or developmental biology.^19^ In the spirit of avoiding what the subcommittee terms ‘stem cell exceptionalism’,^20^ animal research committees assessing chimera experiments should build upon (and their approach should remain broadly consistent with) common animal research review standards.^21^

The ISSCR does suggest some modest departures from existing animal research review processes. However, such departures aim to remain true to the underlying spirit of common animal research principles; they are not designed to address moral status issues. For example, the ISSCR recommends monitoring certain categories of chimeric animals for behavioral abnormalities, including in cases where there is ‘significant potential [for the research] to create some aspect suggestive of human cognition, self‐awareness, behavior or behavioural pathology’.^22^ However, the aim of such monitoring is merely to ensure the humane protection of animal subjects, as per general animal research principles; there is no suggestion that research with such animals should not proceed. Altered and enhanced cognitive capacities are treated as a practical challenge for implementing existing animal welfare standards, not an issue of moral status.

Similarly, in the case of human‐monkey brain chimeras—an area of chimera research widely thought to raise particularly serious moral status issues—the ISSCR white paper offers only practical advice about handling and housing nonhuman primates. There is guidance on how to manage bite and scratch risks to animal care staff and scientists, reduce animal distress, and minimise distress behaviours (such as self‐inflicted wounding) that might complicate the task of assessing the effects of chimerism on the animal.^23^ However, there is again no discussion of how the potential development of enhanced cognitive capacities would affect the permissibility of the research.

The second broad category of chimera research addressed in the guidelines is research with chimeric embryos and fetuses. In this case, the ISSCR recommends a specialised scientific and ethics review process if the embryo or fetus is developed in utero. Specific recommendations include that such experiments proceed only if no alternative models are available, that chimeric embryos not be gestated for longer than necessary to achieve the scientific aim, and that researchers attempt to restrict chimerism to specific organ systems. 24 In cases where chimeric animals are brought to term, the ISSCR recommends utilising existing institutional animal research ethics committee oversight, as per its recommendations for postnatal chimeric animals.^25^ As described above, these recommendations aim merely to adapt and apply existing animal ethics principles, and therefore do not address worries about moral status enhancement.

The ISSCR White Paper describes its approach as a response to ‘potential concerns around the possibility of significant or meaningful enhancement of animal cognition by human cells’, albeit a response that ‘tries to avoid giving undue influence to unsupported, imagined possibilities’.^26^ Yet rather than striking a middle path between these two concerns, the ISSCR ultimately treats live‐born chimera as a novel subset of animal research that may require some tweaks to existing animal welfare practices but does not raise any substantive new ethical issues.

Beyond its recommendations for the oversight of prenatal and postnatal chimera research, the ISSCR calls for the stem cell research community to avoid ‘implying human cognitive abilities, human consciousness of self‐awareness’ when communicating with the public about human–animal chimeras, since this might ‘sow[] doubts about the legitimate nature of such research’.^27^ In effect, scientists are encouraged to communicate about chimera research in ways that undercut public concerns about (and debate regarding) chimeric animals' moral status.

It is worth acknowledging that these recommendations are open to change in future revisions of the ISSCR guidelines. The subcommittee aimed to provide guidance for the next 5–10 years, a timespan in which the subcommittee believes the most ethically worrisome scenarios (such as the ‘generation of laboratory animals with human‐like cognitive traits’) are unlikely to occur.^28^ If these troubling applications remain far on the horizon, then perhaps for now a permissive approach is appropriate. Yet as I show below, the ISSCR subcommittee also offers two *additional* arguments in defence of its permissive approach, and these arguments seem to be consistent with allowing the most ethically troubling forms of chimera research to proceed once they become feasible. In other words, certain strands of argument used by the ISSCR subcommittee (and developed within the bioethical literature on human–animal chimeras that the subcommittee draws heavily from) would seem to commit the ISSCR to a highly permissive approach not just in the present, but also indefinitely into the future. It is these strands of argument that are the focus of this paper.

In short, the 2021 ISSCR Guidelines afford chimeric animals no greater protections than other research animals. This is not to say that chimeric animals would lack protection altogether. They would still be subject to existing principles of animal research ethics, such as the 3Rs.^29^ These hold that animal models should be **R**eplaced where possible, the number of animals used per experiment **R**educed to a minimum, and scientific techniques **R**efined to minimise animal suffering. Importantly, however, the Three Rs are limited to minimising *unnecessary* animal suffering in the pursuit of valid scientific objectives; they do not weigh the value of animal welfare against scientific objectives.^30^ The Three Rs (and other common animal research review standards) thus treat research animals as if they have negligible moral status.^31^

## 4 IS THE ISSCR RIGHT TO DISMISS MORAL STATUS CONCERNS?

Like many of the sources described above, the White Paper holds that humans' moral status is grounded in our sophisticated cognitive capacities, including human intellectual processing capabilities and self‐consciousness. So why do the ISSCR recommendations depart so sharply from other reports and discussions on human–animal neurological chimeras?

One initial reason, flagged above, is that the ISSCR subcommittee bracketed off ethical concerns about the development of human‐like cognitive traits since in the subcommittee's view there is little chance *in the near future* of producing chimeric animals of sufficient cognitive complexity to elevate their moral status beyond that of other research animals.^32^ I can see three possible responses to this view. The first would hold that concerns about cognitive enhancement are already more serious than they appear. In 2013, a ground‐breaking study investigated some cognitive abilities of chimeric mice whose brains contained human glial cells. These mice outperformed their wild‐type counterparts on tests of learning, memory, and fear conditioning.^33^ One could perhaps argue, on the basis of this and other evidence, that the cognitive enhancement of chimeric animals in the near future is not farfetched. The second response would hold that *even if* cognitive enhancement is not already a live concern, it is difficult to predict the pace of scientific progress, and thus how soon moral status concerns might become relevant. The rate of scientific progress is often surprising, even for scientists at the center of research in a particular field. Indeed, in 1901—only 2 years before the Wright brothers achieved the first successful flight of a heavier‐than‐air flying machine—Wilbur Wright announced that powered flight was 50 years away.^34^ Current estimates of the progress of human–animal chimera research might be similarly off‐target. The third response would hold that even if substantial cognitive enhancement is a future concern, there is value in identifying moral limits for such research *now*. Doing so will help protect against inadvertent wrongdoing further in the future, and will further enable scientists to show how their current experiments fall within ethical boundaries (and thus help preserve public trust in a controversial but important area of research.)

We can, however, leave these issues to one side. The ISSCR's reasoning does not stop with the claim that substantial cognitive enhancement of chimeric animals is unlikely to occur in the near future. They also offer two additional arguments aimed at defusing these concerns—and such arguments support an unusually permissive approach to human–animal chimera research not just now, but also in a possible distant future where scientists are able to create human–animal brain chimeras that *can* develop highly sophisticated cognitive capacities. Regardless of what one makes about the likelihood of chimeric animals developing such capacities in the next 5–10 years, these two additional (and more fundamental) arguments ought to concern us.

Drawing on earlier work by Insoo Hyun (who is also the first author of the white paper),^35^ the ISSCR subcommittee gives two reasons to doubt that human–animal chimeras would not develop traits that confer moral status on a par with humans.^36^ The first is that sophisticated cognitive capacities and self‐consciousness develop in humans only after years of attentive child‐rearing. By contrast, chimeric research animals would not be raised under the necessary social and nurturing conditions, and in many cases would not reach full maturity. Second, regardless of the degree to which human cells contribute to a chimeric animal's brain, there will presumably be differences in brain size and structure ‐ and even if a fully human brain were to develop, it would be embodied in an animal whose sensory input and motor output systems are very different from those of a normal human. Accordingly, a human brain in the body of (say) a pig or a monkey would not develop a normal human mind.

The following two sections address these two arguments, beginning with the second. First, however, it is worth noting that Insoo Hyun adopts a highly demanding threshold for full moral status (or moral status of a degree that would render a being's use in animal research unethical.) According to Hyun, human beings' moral status is grounded in a distinctly human form of self‐consciousness involving propositional beliefs, which Hyun holds can likely only arise in beings with a capacity for language.^37^ (The ISSCR White Paper likewise references self‐consciousness but does not specify what kind of self‐consciousness is meant. However, given that the ISSCR subcommittee cites Hyun in support of its claims about the moral relevance of self‐consciousness, they presumably have a similar account of self‐consciousness in mind.) It might be asked whether this threshold is too high. Despite lacking language, chimpanzees and other Great Apes are sometimes argued to have full moral status by dint of their rationality, autonomy, and other complex capacities,^38^ and many jurisdictions prohibit harmful experimentation with these animals for this reason.^39^ Other philosophers argue that self‐consciousness is insignificant to moral status,^40^ and instead ground moral status in the nature and strength of a being's interests.^41^ It is not obvious why chimeric animals should be required to achieve *human* forms of self‐consciousness before being granted serious moral consideration.^42^ I will, however, leave this issue to one side. Even if the ISSCR is right to set a highly demanding threshold for full moral status, their reasons for dismissing moral status concerns have deeply troubling implications.

### 4.1 Can full moral status emerge only in a normal human brain and body?

What should we make of the claim that morally relevant capacities can emerge only in a human brain that is housed in a human body? This claim is speculative; there has been little research examining the cognitive capacities of human–animal neurological chimeras (and especially chimeric nonhuman primates and large mammals), for the simple reason that relatively few have so far been created. But perhaps more troubling for this claim is that there is a great deal of diversity in the brains and bodies of humans, including humans who are widely thought to have full moral status. Consider the striking case, first reported in 2007, of a 44‐year‐old whose brain formed in a thin layer around a large fluid‐filled chamber, with a reduction of brain volume of more than 50%–75%. Despite this abnormality, the man's neurological development was otherwise normal, and he had a successful career as a civil servant.^43^ The capacities that undergird humans' moral status have already been realised in brains that differ, sometimes quite significantly, from those of a normal human.

What of the differences between the body (and sensory apparatuses/motor output systems) of a human and that of, say, a pig or a monkey? The difficulty with this claim is that human bodies (and their sensory apparatuses) are already greatly varied. Some humans lack the sense of sight or hearing; others are born with missing and malformed limbs. Not all humans live in a ‘normal’ human body. Thankfully, these differences are not usually thought to undermine the moral status of persons with physical disabilities.^44^ Since morally relevant cognitive capacities already develop across a wide variety of human bodies, it is unclear why they could not also develop in the bodies of other animals.

### 4.2 Can moral status concerns be addressed by preventing chimeric animals from developing complex cognitive capacities?

The ISSCR white paper also flags that morally relevant capacities such as self‐consciousness rely, even in humans, on years of child‐rearing under narrow social and nurturing conditions. Unlike human infants, chimeric animals would not be raised under such conditions; accordingly, they might be unable to develop morally relevant cognitive capacities, even if they could theoretically realise them under different conditions. As Insoo Hyun has put the point elsewhere:

[S]elf‐consciousness is a complex mental faculty that takes years to emerge in human infants under the nurturing conditions of our everyday socialisation processes. Chimeric research animals will not be raised within the ‘bosom of society’ … and thus, regardless of their level or nature of chimerism, will not have the supporting conditions necessary to facilitate the emergence of self‐consciousness.^45^

This argument presupposes that moral status should be tied to the capacities a being actually has (rather than those it could potentially develop.) This view, while not implausible, has highly controversial implications. Among other things, it can be used to defend infanticide, as well as certain forms of child abuse and neglect. These are implications that I think many people will reject.

Consider, first, what this view of moral status entails for human infants. Like research animals, infants lack many capacities that are thought to undergird full moral statuses, such as language and complex forms of self‐consciousness. If chimeric animals should not be treated as if they have full moral status *unless and until* they develop morally relevant capacities, then presumably the same would be true of human infants. Both infants and research animals would have a similarly slim degree of moral status.

This view does have its defenders. Some philosophers argue that infants lack any right to life (among other moral rights) until they develop self‐consciousness. Accordingly, practices like infanticide are sometimes thought not to be serious moral wrongs, given infants' lack of full moral status.^46^ Despite the philosophical arguments that have been offered in its defence, many people reject this view. Those who reject this view in relation to human infants should likewise reject it in relation to human–animal chimeras. It, therefore, seems a tenuous basis for global stem cell policy.

There is a further implication of Hyun and the ISSCR subcommittee's argument which is even more troubling. This is linked to the claim that moral status concerns can be nullified by raising chimeric animals under conditions that prevent them from developing the requisite cognitive abilities. If it is permissible to curtail chimeric animals' moral status by raising them under conditions inimical to the development of self‐consciousness and higher‐order intellectual processing abilities, then it seems it should also be permissible to curtail the moral status of human children in the same way.

This possibility is not entirely theoretical. Children who are deprived of language input during childhood (due, e.g., to extreme neglect) may never be able to learn to arrange words to form sentences.^47^ Since Hyun's view of full moral status presupposes a capacity for language, such children would presumably lack the status of ‘normal’ humans.

Even more troublingly, this view of moral status is consistent with choosing to deliberately raise children that would never be considered to have a greater moral status than a research animal by limiting their exposure to language in their early years. Like human–animal chimeras, this cohort of children could theoretically form an ideal platform for toxicity testing, studying human development, and various forms of brain research. (Indeed, since they are *wholly* human, there are scientific reasons to *prefer* the use of such children over the use of chimeric animals.) I suspect, however, that few people would endorse this proposal. Many will have the strong intuition that it is wrong to raise human children under conditions that prevent self‐consciousness or other morally relevant cognitive capacities from developing.^48^ If this vi is correct, then we cannot circumvent concerns about a being's moral status by raising them under conditions that prevent self‐consciousness or other morally relevant capacities from emerging.

## 5 CONCLUSION

The ISSCR's 2021 guidelines for stem cell research hold that moral status concerns should not constrain human–animal chimera research. The ISSCR's reliance on existing animal ethics principles places it at odds with many existing analyses of the ethics of human–animal chimera research, including those enumerated in reports by major research organisations. Moreover, the ISSCR's dismissal of moral status concerns relies on philosophical claims that are deeply controversial and have highly unpalatable implications for our treatment of human infants and children. These claims are too tenuous and controversial to underpin global guidelines for an ethically complex area of research.

## CONFLICT OF INTEREST

The author declares no conflict of interest.

## Footnotes

1 Lovell‐Badge, R. (2021). Stem‐cell guidelines: Why it was time for an update. *Nature*, *593*(7860), 479.

2 Hyun, I., Taylor, P., Testa, G., Dickens, B., Jung, K. W., McNab, A., Robertson, J., Skene, L., & Zoloth, L. (2007). Ethical standards for human‐to‐animal chimera experiments in stem cell research. *Cell Stem Cell*, *1*(2), 159–163.

3 Koplin, J. J., & Savulescu, J. (2019). Time to rethink the law on part‐human chimeras. *Journal of Law and the Biosciences*, *6*(1), 37–50.

4 DeGrazia, D. (2008). Moral status as a matter of degree? *The Southern Journal of Philosophy*, *46*(2), 181–198.

5 Jaworska, A., & Tannenbaum, J. (2021). The grounds of moral status. In E. N. Zalta (Ed.), *The Stanford Encyclopedia of Philosophy* (Spring 2021). Metaphysics Research Lab, Stanford University. https://plato.stanford.edu/archives/spr2021/entries/grounds-moral-status/

6 Greely, H. T. (2021). Human brain surrogates research: The onrushing ethical dilemma. *The American Journal of Bioethics*, *21*(1), 34–45, p. 34.

7 Devolder, K., Yip, L. J., & Douglas, T. (2020). The ethics of creating and using human‐animal chimeras. *ILAR Journal*, *60*(3), 434–438; Koplin, J. J. (2019). Human‐animal chimeras: The moral insignificance of uniquely human capacities. *Hastings Center Report*, *49*(5), 23–32; Koplin & Savulescu, op. cit. note 3; Eberl, J. T., & Ballard, R. A. (2009). Metaphysical and ethical perspectives on creating animal‐human chimeras. *The Journal of Medicine and Philosophy*, *34*(5), 470–486; Streiffer, R. (2007). At the edge of humanity: Human stem cells, chimeras, and moral status. *Journal of Philosophical Research*, *32*(Suppl.), 63–83; Karpowicz, P., Cohen, C. B., & van der Kooy, D. (2005). Developing human‐nonhuman chimeras in human stem cell research: Ethical issues and boundaries. *Kennedy Institute of Ethics Journal*, *15*(2), 107–134.

8 Hagan‐Brown, A., Favaretto, M., & Borry, P. (2017). Newspaper coverage of human‐pig chimera research: A qualitative study on select media coverage of scientific breakthrough. *Xenotransplantation*, *24*(4), e12317.

9 National Institutes of Health. (2017). *Frequently asked questions on chimera proposal*. Office of Science Policy. https://osp.od.nih.gov/wp-content/uploads/QA_Chimera_Policy_updated_1_Feb_2017.pdf

10 National Academy of Sciences, Engineering and Medicine (NASEM). (2021). *The emerging field of human neural organoids, transplants, and chimeras: Science, ethics, and governance*. National Academies Press (US). http://www.ncbi.nlm.nih.gov/books/NBK569428/

11 Ibid: 61.

12 Ibid: 100–192.

13 Academy of Medical Sciences. (2011). *Animals containing human material*. The Academy of Medical Sciences, §3.4.

14 Ibid: §5.6.2.

15 Ibid: §8.2.

16 Cabrera Trujillo, L. Y., & Engel‐Glatter, S. (2015). Human–animal chimera: A neuro driven discussion? Comparison of three leading European research countries. *Science and Engineering Ethics*, *21*(3), 595–617.

17 Hyun, I., Clayton, E. W., Cong, Y., Fujita, M., Goldman, S. A., Hill, L. R., Monserrat, N., Nakauchi, H., Pedersen, R. A., Rooke, H. M., Takahashi, J., & Knoblich, J. A. (2021). ISSCR guidelines for the transfer of human pluripotent stem cells and their direct derivatives into animal hosts. *Stem Cell Reports*, *16*(6), 1409–1415.

18 International Society for Stem Cell Research (ISSCR). (2021). *Guidelines for stem cell research and clinical translation*. https://www.isscr.org/docs/default-source/all-isscr-guidelines/2021-guidelines/isscr-guidelines-for-stem-cell-research-and-clinical-translation-2021.pdf?sfvrsn=979d58b1_4

19 Hyun, I., et al., op. cit. note 17, p. 1411; ISSCR, op. cit. note 18, §2.2.1.1.

20 Hyun, I., et al., op. cit. note 17, p. 1410.

21 Ibid: 1412.

22 Ibid: 1413.

23 Ibid: 1414.

24 Ibid: 1412.

25 Ibid.

26 Ibid.

27 ISSCR, op. cit. note 18, §4.1.

28 Hyun, I., et al., op. cit. note 17, p. 1410.

29 Ibid: 1412.

30 Tannenbaum, J., & Bennett, B. T. (2015). Russell and Burch's 3Rs then and now: The need for clarity in definition and purpose. *Journal of the American Association for Laboratory Animal Science*, *54*(2), 120–132.

31 This is the key reason why it would seem outrageous to govern human subject research according to the Three R's or other established animal research principles—because such principles are entirely ill‐suited to beings with full or substantial moral status (a degree of moral status which normal human adults certainly have.)

32 Hyun, I., et al., op. cit. note 17, p. 1410.

33 Han, X., Chen, M., Wang, F., Windrem, M., Wang, S., Shanz, S., Xu, Q., Oberheim, N. A., Bekar, L., Betstadt, S., Silva, A. J., Takano. T., Goldman, S. A., & Nedergaard, M. (2013). Forebrain engraftment by human glial progenitor cells enhances synaptic plasticity and learning in adult mice. *Cell Stem Cell*, *12*(3), 342–353.

34 Dryden, H. L. (1960). Penrose memorial lecture. Prospects for space travel. *Proceedings of the American Philosophical Society*, *104*(5), 474–484, p. 474.

35 Hyun, I. (2016). What's wrong with human/nonhuman chimera research? *PLOS Biology*, *14*(8), e1002535; Hyun, I. (2013). *Bioethics and the future of stem cell research*. Cambridge University Press.

36 Hyun, I., et al., op. cit. note 17, p. 1412.

37 Hyun, I. (2019). Ethical considerations for human–animal neurological chimera research: Mouse models and beyond. *The EMBO Journal*, *38*(21), e103331; Hyun, I. (2015). From naïve pluripotency to chimeras: A new ethical challenge? *Development*, *142*(1), 6–8.

38 Andrews, K., Comstock, G. L., Crozier, G. K. D., Donaldson, S., Fenton, A., John, T. M., Johnson, L. S. M., Jones, R. C., Kymlicka, W., Meynell, L., Nobis, N., Pena‐Guzman, P., & Sebo, J. (2018). *Chimpanzee rights: The philosophers' brief*. Routledge.

39 Aguilera, B., Perez Gomez, J., & DeGrazia, D. (2021). Should biomedical research with great apes be restricted? A systematic review of reasons. *BMC Medical Ethics*, *22*(1), 15.

40 Shepherd, J. (2017). The moral insignificance of self‐consciousness. *European Journal of Philosophy*, *25*(2), 398–415.

41 Koplin, op. cit. note 7; Shepherd, J. (2018). *Consciousness and moral status*. Routledge; DeGrazia, D. (1996). *Taking animals seriously: Mental life and moral status*. Cambridge University Press.

42 Interestingly, the ISSCR guidelines prohibit the use of great and lesser ape species in several kinds of chimera research. One common justification for prohibiting such research is because apes are commonly thought to have a high degree of moral status (rendering invasive research morally impermissible) *despite* their lack of human‐like language abilities. Neither the ISSCR guidelines nor the White Paper directly endorse such views, instead noting merely that the use of apes for invasive research is prohibited in much of the world. However, if apes have similar moral status to humans, this would cut against the claim that a capacity for language is necessary for an animal to have sufficient moral status that it should be excluded from chimera research.

43 Feuillet, L., Dufour, H., & Pelletier, J. (2007). Brain of a white‐collar worker. *The Lancet*, *370*(9583), 262; New Scientist. (2007). *Man with tiny brain shocks doctors*. https://www.newscientist.com/article/dn12301-man-with-tiny-brain-shocks-doctors/

44 Whether severe cognitive disabilities can affect moral status is more controversial. See: McMahan, J. (2002). *The ethics of killing: Problems at the margins of life*. Oxford University Press.

45 Hyun, op. cit. note 37, p. 7. The ISSCR White Paper likewise hints at this argument. Citing work by Insoo Hyun, it holds that ‘complex mental traits [such as self‐consciousness] are not biologically assured even in infant brains that are 100% human, absent the experience of social and nurturing conditions of child‐rearing over a time span of years’. See: Hyun, I., et al., op. cit. note 17, p. 1412.

46 Giubilini, A., & Minerva, F. (2013). After‐birth abortion: Why should the baby live? *Journal of Medical Ethics*, *39*(5), 261–263. Singer, P. (2011). *Practical ethics*. Cambridge University Press; Tooley, M. (1972). Abortion and Infanticide. *Philosophy & Public Affairs*, *2*(1), 37–65.

47 Friedmann, N., & Rusou, D. (2015). Critical period for first language: The crucial role of language input during the first year of life. *Current Opinion in Neurobiology*, *35*, 27–34.

48 Alternatively, it might be the case that the bar for full moral status should be set much lower than the development of (sophisticated forms of) self‐consciousness, so that human infants and children would possess it from a very young age. In this case, the problem with the ISSCR's approach is in where it sets the threshold for full moral status—a possibility we flagged earlier in the paper.

